# Autologous platelet concentrates as adjuvant in the surgical management of medication‐related osteonecrosis of the jaw

**DOI:** 10.1111/prd.12608

**Published:** 2024-09-30

**Authors:** Francesco Bennardo, Selene Barone, Alessandro Antonelli, Amerigo Giudice

**Affiliations:** ^1^ School of Dentistry Magna Graecia University of Catanzaro Catanzaro Italy

**Keywords:** L‐PRF, ONJ, osteonecrosis, platelet concentrates, PRGF, PRP, review

## Abstract

Medication‐related osteonecrosis of the jaw (MRONJ) is an infectious side effect associated with bisphosphonates and monoclonal antibodies (denosumab, immune modulators, and antiangiogenic medications). Adjunctive therapies for the surgical management of MRONJ include autologous platelet concentrates (APCs). These APCs serve as a source of various cells and growth factors that aid tissue healing and regeneration. This review evaluated the use of platelet‐rich plasma (PRP), plasma‐rich in growth factors (PRGF), and leukocyte‐ and platelet‐rich fibrin (L‐PRF) as adjuvant therapies for the surgical management of MRONJ by conducting analyses on the results of 58 articles. Compared to surgical treatment alone, the application of PRP and L‐PRF after surgery appears to increase healing in the management of patients with MRONJ. No studies have reported unhealed lesions as a result of surgical treatment of MRONJ with PRGF application or compared it with surgical treatment alone. The overall results of this review have shown favorable healing rates of MRONJ lesions managed with the application of APCs after surgical treatment; however, significant methodological limitations may limit the scientific evidence supporting their use. Further randomized controlled trials with strict criteria are needed to establish the extent to which APCs can improve wound healing and quality of life in patients with MRONJ requiring surgical treatment.

## 1 INTRODUCTION

Medication‐related osteonecrosis of the jaw (MRONJ) is an infectious side effect of bone‐modifying agents (BMA), such as bisphosphonates or denosumab, or anti‐cancer drugs (ACD), including immune modulators or antiangiogenic medications. The American Association of Oral and Maxillofacial Surgeons (AAOMS) defined MRONJ as the persistent exposure of bone within the oral cavity (or bone that can be probed through an extraoral or intraoral fistula) for at least 8 weeks and progressive involvement of the jaws. MRONJ occurs in patients who undergone BMA or ACD without metastatic disease of the jaw or history of radiotherapy to the head and neck region.^1^

The MRONJ‐related BMA include zoledronate, pamidronate, alendronate, risedronate, ibandronate, neridronate, clodronate, tiludronate, etidronate, denosumab, and romosozumab, while the MRONJ‐related ACD include bevacizumab, aflibercept, sunitinib, sorafenib, cabozantinib, regorafenib, axitinib, temsirolimus, and everolimus. Ipilimumab, adalimumab, etanercept, tocilizumab, methotrexate, rituximab, imatinib, and lenvatinib have been reported to be potentially associated with MRONJ; however, no evidence has been reported of such a relationship.^2^, ^3^, ^4^, ^5^, ^6^

Much debate persists regarding the pathophysiology of MRONJ. However, the mechanisms underlying its pathogenesis include altered bone remodeling, infectious and inflammatory processes, innate or acquired immune dysfunction, angiogenesis inhibition, genetic factors, and local drug toxicity.^7^, ^8^ Experimental and human studies have indicated that the combination of BMA with infection or inflammation is both necessary and sufficient to trigger MRONJ. Nevertheless, as our understanding of the subject grows, it has become increasingly evident that MRONJ is a multifactorial condition, and multiple hypotheses may elucidate its pathophysiology.^1^

MRONJ can be induced by dentoalveolar surgery or develop spontaneously in the jaw. In both cases, local risk factors include periodontal disease, periapical pathology, and denture use. Prevention and control of these risk factors are important to avoid or reduce the risk of MRONJ in patients undergoing BMA or ACD.^1^, ^9^ Almost every patient with MRONJ shows a loss of the oral mucosa around the infected bone, which could also be related to the toxicity of BMA and ACD in soft tissues. Therefore, soft tissue healing may play a role in the surgical management of MRONJ lesions.^10^

Adjunctive therapies for the surgical management of MRONJ include ozone therapy, laser treatment, hyperbaric oxygen therapy, and autologous platelet concentrates (APCs).^9^, ^11^

**FIGURE 1:**
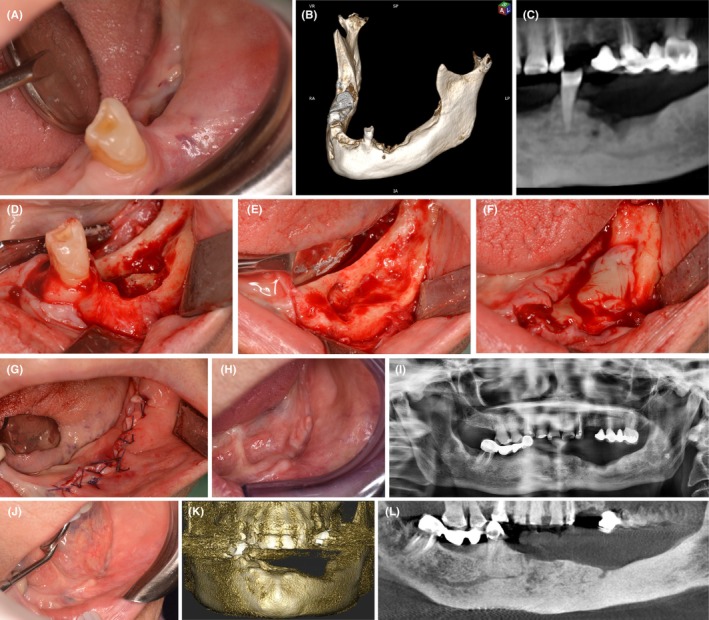
(A) Intraoral pre‐operative photograph of a patient with MRONJ in the left mandible. (B) Three‐dimensional reconstruction of CBCT images of the patient. (C) Panoramic dental reconstruction of CBCT images of the patient. (D) Intraoperative picture of MRONJ lesion after flap elevation. (E) Intraoperative picture after necrotic bone removal and tooth extraction. (F) L‐PRF membranes application to cover bone defect. (G) Tension‐free wound closure with resorbable suture. (H) Healing at 6 months after surgery. (I) Orthopantomography at 6 months after surgery. (J) Healing at 12 months after surgery. (K) Three‐dimensional reconstruction of CBCT images of the patient (mandible) at 12 months after surgery. (L) CBCT image of the patient at 12 months after surgery.

APCs are produced with the patient's blood and contain high concentrations of various growth factors (GFs) and cytokines released by platelets. APCs play a key role in stimulating and expediting wound healing, and promoting the regeneration of both hard and soft tissues. By harnessing the combined potential of platelets and fibrin matrices, APCs serve as a biotechnological solution capable of providing sustained release of GFs essential for tissue repair processes. These mechanisms include chemotaxis, antimicrobial effects, angiogenesis, cell proliferation, cell differentiation, and tissue remodeling.^12^ The use of different APCs in the management of MRONJ has been investigated over the past 15 years.^13^, ^14^, ^15^ In 2005, Curi et al.^16^ reported the use of platelet‐rich plasma (PRP) and its possible effect on the surgical management of MRONJ. In 2012, Mozzati et al.^17^ reported the successful use of plasma‐rich in growth factors (PRGF) in 32 patients with MRONJ who were managed surgically. In 2013, Soydan and Uckan^18^ first described the surgical management of MRONJ using leukocyte‐ and platelet‐rich fibrin (L‐PRF) membranes placed at the surgical site before suturing. These reports show promising results for the application of APCs in the surgical management of MRONJ (Figure 1A–L).

This article aimed to provide a comprehensive overview of the use of APCs as an adjuvant therapy in the surgical management of MRONJ, including wound healing and patient‐reported outcome measures (PROMs), achieved through an analysis of all scientific literature available on the topic.

## 2 EVIDENCE SEARCH METHODOLOGY

An electronic search was performed to gather evidence of the benefits of APCs in the treatment of MRONJ, using the Web of Science, Scopus, and Medline (using PubMed). Articles published until June 30, 2023, were included. The keywords used were “osteonecrosis”, “platelet concentrate”, “platelet‐rich plasma” and “platelet‐rich fibrin.”

### 2.1 Inclusion and exclusion criteria

No restrictions were imposed on the study design. The following inclusion criteria were applied: (1) original publications in the English language; (2) human studies; (3) patients diagnosed with MRONJ (in accordance with AAOMS) and undergoing treatment with BMA or ACD for malignancy or metabolic diseases; (4) use of APCs (PRP, PRGF, and L‐PRF); and (5) presence of outcome variables in the article.

The following exclusion criteria were applied: (1) non‐systematic reviews of the literature, doctoral theses, editorials, and abstracts; (2) in vitro studies; and (3) animal studies. The reference lists of the literature reviews were analyzed to search for additional publications.

### 2.2 Selection of studies and data extraction

The selected articles included case reports, case series, prospective studies, retrospective studies, clinical trials, and systematic reviews. Two authors (FB and SB) independently conducted electronic searches of all databases, and discrepancies were resolved through a consensus meeting with a third author (AG). Data extraction was performed independently by two authors (AA and FB). Any disagreements were subjected to further evaluation with a third author (AG). The studies were categorized according to the type of platelet concentrate used (PRP, PRGF, or L‐PRF).

In addition to the type of study, the following variables were extracted from the articles: sample size, age, sex, etiology, type, dosage, duration of drug treatment, comorbidities, AAOMS staging (Table 1), jaw receiving treatment (maxilla and/or mandible), follow‐up duration, and outcome variables (including recurrences and complications).

**TABLE 1:** MRONJ staging based upon AAOMS classification.

Patients at‐risk	No bone exposure, no symptoms
Stage 0	No bone exposure Non‐specific symptoms pain (teeth, jaws, sinus, and/or temporomandibular joint) not explained by an odontogenic cause altered neurosensory function Clinical findings loosening of teeth not explained by chronic periodontal disease intraoral or extraoral swelling Radiographic findings bone loss not related to periodontal disease sclerotic pattern of trabecular bone altered socket healing osteosclerosis
Stage I	Bone exposure in the oral cavity (or bone that can be probed through an extraoral or intraoral fistula) for a minimum of 8 weeks No specific symptoms No evidence of inflammation or infection Radiographic findings as Stage 0
Stage II	Stage I characteristics and specific symptoms and evidence of inflammation or infection
Stage III	Stage II characteristics and one or more of the following signs: exposed basal bone or osteolysis extending to basal bone or maxillary sinus oral antral fistula/oral‐nasal fistula extraoral fistula bone fracture

The outcome variables of the MRONJ treatment studies were classified into three categories, according to previous descriptions^15^, ^19^, ^20^, ^21^:

- complete healing: complete remission with complete mucosal coverage over the previously involved area;
- partial healing: improvement in the disease (quality of life and stage) without complete mucosal coverage of the exposed bone;
- no healing: no improvement in clinical signs and symptoms during observation or disease progression.

### 2.3 Results

A total of 899 articles were retrieved using an electronic search, and two additional articles were obtained via a manual search. After removing duplicates, the authors screened 346 titles and abstracts and selected 97 articles for full‐text screening. Finally, 58 articles were included in this review. Data from case reports with fewer than five patients were not analyzed.

## 3 APCs IN THE TREATMENT OF MRONJ

### 3.1 Summary

A total of 538 patients presenting with 557 lesions who underwent surgery with APC application were recruited from the included studies. Considering the outcomes at follow‐up visits, APC treatment showed complete healing in 480 lesions (86.2%), partial healing in 32 lesions (5.7%), and no healing in 45 lesions (8.1%).

Among the included studies, 37 patients who underwent surgery without APC application were recruited as controls. Forty‐two lesions were treated. Considering the outcomes at follow‐up visits, treatment without APCs showed complete healing in 34 lesions (80.9%), and no healing in eight lesions (19.1%).

## 4 PRP IN THE MANAGEMENT OF MRONJ

Fifteen studies related to PRP adjuvants in the surgical treatment of MRONJ were included (Table 2), comprising 10 case report and case series, three retrospective studies, and two prospective studies.

**TABLE 2:** PRP in the management of MRONJ: Studies included.

Study type	Number	References
Case report and case series	10	Adornato et al.^22^ Antonini et al.^23^ Bernardi et al.^24^ Cetiner et al.^25^ Coviello et al.^26^ Curi et al.^16^ Curi et al.^27^ Lee et al.^28^ Merigo et al.^29^ Vairaktaris et al.^30^
Retrospective studies	3	Longo et al.^31^ Martins et al.^32^ Mathias Duarte et al.^33^
Prospective studies	2	Bocanegra‐Perez et al.^34^ Mauceri et al.^35^

*Note*: Data related to the case reports (<5 patients included) are available in Tables S1 and S2.

All data concerning the studies are presented in Tables 3 and 4. A total of 130 patients who underwent surgery with PRP were recruited for these studies, and a total of 135 lesions were treated using this protocol. Considering the outcomes at follow‐up visits (up to 94 months), PRP treatment resulted in complete healing of 113 lesions (83.7%), partial healing of 12 lesions (8.9%), and no healing of 10 lesions (7.4%). No adverse effects were observed. It is important to emphasize that there was no uniformity in the description of the criteria for a partial or negative response to treatment (including recurrence).

**TABLE 3:** PRP in the management of MRONJ: General features of the studies.

References	Patients number	Age (mean; range; years)	Gender (M/F)	Disease	Medication administered	Duration (mean, range or other)	Habits, comorbidities, other medications (if reported)
Adornato et al.^22^	12	63.9 ± 11.3 (43–83)	4/8	Breast cancer 8 Prostatic cancer 1 Multiple myeloma 3	Z 8 P 4	At least 1 year	Smoking 2
Bocanegra‐Pérez et al.^34^	8	66.3 ± 9.6	2/6	Multiple myeloma 4 Breast cancer 2 Osteoporosis 2	*Cancer* Z 3 Z + P 2 Z + P + C 1 *MBD* A 2	–	Diabetes 6 Corticosteroids 5
Curi et al.^27^	25	60.7 ± 10.1	5/20	Breast cancer 14 Prostate cancer 4 Multiple myeloma 7	Z 21 P 4	2.3 years (range 1–7 years)	Chemotherapy 24 Corticosteroids 7
Longo et al.^31^	34	59 (37–81) (data referred to 72 patients with MRONJ)	12/60 (data referred to 72 patients with MRONJ)	Breast cancer 54 Lung cancer 8 Prostatic cancer 9 Multiple myeloma 1 (data referred to 72 patients with MRONJ)	A 2 P 22 Z 48 (data referred to 72 patients with MRONJ)	4–62 m (data referred to 72 patients with MRONJ)	–
Martins et al.^32^	14	55.6 (42–90)	5/9	Breast cancer 7 Lung cancer 1 Prostatic cancer 5 Multiple myeloma 1	Z 10 P 4	22.57 m (range 8–48 m)	Chemotherapy 13 Corticosteroids 8
Mathias Duarte et al.^33^	6	68.3 (54–84)	1/5	Breast cancer 4 Prostatic cancer 1 Osteoporosis 1	*Cancer* Z 5 *MBD* A 1	–	Cardiovascular disease 1 Diabetes 1 Hypertension 1
Mauceri et al.^35^	10	75.2 ± 5.9	3/7	Breast cancer 3 Prostate cancer 3 Multiple myeloma 4	Z 9 Z + I 1	31.8 ± 25.76 m	Chemotherapy 5 Corticosteroids 2 Diabetes 2 Hypertension 6 Osteoporosis 5 Rheumatoid arthritis 1 Smoking 2
Merigo et al.^29^	21	72.6 (60–85)	5/16	Breast cancer 7 Kidney cancer 1 Pancreas cancer 1 Prostate cancer 2 Osteoporosis 8 Osteoporosis + rheumatoid arthritis 1 Rheumatoid arthritis 1	*Cancer* Z 8 Z + S 3 *MBD* A 10	5–164 m	Arhythmia 1 Corticosteroids 6 Diabetes 1 Gastritis 1 Hypertension 6 Venous thrombosis 1 Smokers 3

*Note*: Zoledronic acid treatment for cancer patients usually involves administration of 4 mg every 4 weeks. Zoledronic acid treatment for MBD patients usually involves administration of 5 mg once a year. Denosumab treatment for cancer patients usually involves administration of 120 mg every 4 weeks. Denosumab treatment for MBD patients usually involves administration of 60 mg every 6 months.

Abbreviations: A, alendronic acid; B, bevacizumab; BP, bisphosphonate not specified; C, clodronic acid; CR, case reported; D, denosumab; I, ibandronic acid; MBD, metabolic bone disease; n.s, not specified; N/A, not available; P, pamidronic acid; R, risedronic acid; S, sunitinib; Z, zoledronic acid.

**TABLE 4:** PRP in the management of MRONJ: Specific features and outcomes of the studies.

References	Number of lesions	Site	Staging AAOMS (specified if different)	Type of intervention	Antibiotic therapy	Follow‐up	Outcome
Adornato et al.^22^	12	Mandible 8 Maxilla 4	–	Bone resection + PRP	Clindamycin 300 mg × 4/day for 10 days	6 m	10 Complete healing 2 No healing
Bocanegra‐Pérez et al.^34^	10	Mandible 9 Maxilla 1	Stage 2	Bone resection + PRP	–	Average 14 m (range 12–26 m)	Complete healing
Curi et al.^27^	25	Mandible 18 Maxilla 7	3 Stage 1 15 Stage 2 7 Stage 3	Partial bone resection (GA) + PRP	Clindamycin 600 mg i.v. for 7 days	36 m	20 Complete healing 5 No healing
Longo et al.^31^	34	–	1 Stage 1 26 Stage 2 7 Stage 3	Surgical debridement + PRP	Phenoxymethylpenicillin, amoxicillin, amox/clav, or clindamycin with or without metronidazole	6–94 m	32 Complete healing 2 Partial healing
Martins et al.^32^	16	Mandible 12 Maxilla 4	2 Stage 1 9 Stage 2 3 Stage 3	Surgical debridement and sequestrectomy + PRP + laser phototherapy (LPT)	Clindamycin 300 mg or amoxicillin 500 mg for a minimum of 7 days	6 m	15 Complete healing 1 Partial healing
Mathias Duarte et al.^33^	7	Mandible 5 Maxilla 2	Stage 2	Surgical debridement and sequestrectomy + PRP	Clindamycin 300 mg every 6 h	–	3 Complete healing 3 Partial healing 1 No healing
Mauceri et al.^35^	10	Mandible 9 Maxilla 1	SICMF‐SIPMO staging Stage 1B, 6 Stage 2A, 2 Stage 2B, 2	Surgical debridement and sequestrectomy with Er,Cr:YSGG laser device + PRP	Ampicillin/sulbactam 1 g (i.m.) × 2/day + Metronidazole: 500 mg (per os) × 3/day starting 1 day before surgery and for 7 days	15 days 1, 3, 6, 12 m	3 Complete healing 5 Partial healing 2 No healing
Merigo et al.^29^	21	Mandible 6 Maxilla 15	2 Stage 1 15 Stage 2 4 Stage 3	Surgery with Er:YAG laser and piezoelectric devices + PRP	Amox/clav 2 g/day and metronidazole 500 mg/day per os (or clindamycin in case of allergy), starting 3 days before surgery and for at least 2 weeks	9.6 months	20 Complete healing 1 Partial healing

Abbreviations: Amox/clav, amoxicillin + clavulanate; BFP, Buccal fat pad; GA, under general anesthesia; HBO, hyperbaric oxygen therapy; i.m, intramuscular administration; i.v, intravenous administration; N/A, not available.

### 4.1 Comparison between treatments with/without PRP

Among the included studies, Longo et al., Martins et al., and Mathias Duarte et al. retrospectively reported the results of surgical treatment with and without PRP or nonsurgical management of MRONJ in the same cohort of patients. Longo et al. reported the results of the treatment of 72 patients with MRONJ, all initially managed conservatively with systemic antibiotic therapy and local antiseptic rinses. Lesions without improvement underwent surgery, 15 patients received surgical debridement, and 34 patients underwent surgery with PRP application. Among the 15 patients treated with surgery alone, eight showed complete healing and seven showed partial healing. Thirty‐two patients showed complete healing after surgical treatment with PRP and two patients showed only a partial response.^31^ Martins et al. reported the results of 22 patients with 24 MRONJ lesions. Three lesions were managed with non‐surgical medical therapy (systemic antibiotics and local antiseptics), resulting in one complete healing and two partial responses. Sixteen lesions were managed with PRP application and laser phototherapy after sequestrectomy and bone surgery, with complete resolution in 15 lesions and partial response in one lesion. The other five lesions were managed surgically without the application of PRP, resulting in three complete resolutions and two partial healing.^32^

Mathias Duarte et al. reported the results of the treatment of 13 patients with 14 MRONJ lesions. Three lesions were managed with non‐surgical medical therapy (systemic antibiotics and local antiseptics), resulting in two complete resolutions and one negative response. Seven lesions were managed with PRP after surgical resection with complete resolution in three lesions, partial response in three lesions and negative response in one lesion. The other four lesions were managed surgically without the application of PRP, resulting in three partial and one negative response to the treatment.^33^

Considering the aforementioned studies, PRP application after surgery may increase healing in patients with MRONJ.

## 5 PRGF IN THE MANAGEMENT OF MRONJ

Five studies related to PRGF adjuvant therapy in the surgical treatment of MRONJ were included (Table 5): three case reports and two retrospective studies.

**TABLE 5:** PRGF in the management of MRONJ: Studies included.

Study type	Number	References
Case report and case series	3	Anitua et al.^36^ Gil et al.^37^ Pardiñas López et al.^38^
Retrospective studies	2	Mozzati et al.^17^ Sánchez‐Gallego Albertos et al.^39^

*Note*: Data related to the case reports (<5 patients included) are available in Tables S3 and S4.

All the data concerning the studies are presented in Tables 6 and 7. A total of 102 patients who underwent surgery with PRGF were recruited for this study and a total of 102 lesions were treated using this protocol. Considering the outcomes at follow‐up visits (up to 50 months), PRGF treatment resulted in complete healing of 89 lesions (87.3%), and no healing of 13 lesions (12.7%). No adverse effects were observed. It is important to emphasize that there was no uniformity in the description of the criteria for partial or negative response to treatment (including recurrence). However, these studies were too heterogeneous to draw any conclusions.

**TABLE 6:** PRGF in the management of MRONJ: General features of the studies.

References	Patients number	Age (mean; range; years)	Gender (M/F)	Disease	Medication administered	Duration (mean, range or other)	Habits, comorbidities, other medications (if reported)
Mozzati et al.^17^	32	69.7 (44–83)	10/22	Breast cancer 5 Lung carcinoma 4 Multiple myeloma 14 Ovarian carcinoma 3 Prostatic carcinoma 6	Z 26 P 6	37 m	Chemotherapy 4 Corticosteroids 11 Smoking 12
Sánchez‐Gallego Albertos et al.^39^	70	6p <50 years 45p 50–70 years 19p >70 years	12/58	Breast cancer 21 Multiple myeloma 8 Osteoporosis 34 N/A 7	Z 31 D 10 Oral BP 29	5p <6 m 13p 6–12 m 40p >12 m	Hypertension 33 Diabetes 16 Smoking 19 Corticosteroids 18

*Note*: Zoledronic acid treatment for cancer patients usually involves administration of 4 mg every 4 weeks. Zoledronic acid treatment for MBD patients usually involves administration of 5 mg once a year. Denosumab treatment for cancer patients usually involves administration of 120 mg every 4 weeks. Denosumab treatment for MBD patients usually involves administration of 60 mg every 6 months.

Abbreviations: A, alendronic acid; BP, bisphosphonate not specified; D, denosumab; I, ibandronic acid; MBD, metabolic bone disease; N/A, not available; P, pamidronic acid; R, risedronic acid; Z, zoledronic acid.

**TABLE 7:** PRGF in the management of MRONJ: Specific features and outcomes of the studies.

References	Number of lesions	Site	Staging AAOMS (specified if different)	Type of intervention	Antibiotic therapy	Follow‐up	Outcome
Mozzati et al.^17^	32	Mandible 24 Maxilla 8	N/A	Marginal resection surgery with piezoelectric device + PRGF	Amoxicillin 1 g × 2/day from 1 day before the surgery and for 5 days after	48–50 m	Complete healing
Sanchez Gallego‐Albertos et al.^39^	70	Mandible 52 Maxilla 18	4 Stage 1 52 Stage 2 14 Stage 3	Surgical debridement and sequestrectomy + PRGF	N/A	N/A	57 Complete healing (no recurrence) 13 No healing (recurrence)

Abbreviation: Amox/clav, amoxicillin + clavulanate; N/A, not available.

## 6 L‐PRF IN THE MANAGEMENT OF MRONJ

Thirty‐two studies related to L‐PRF adjuvants in the surgical treatment of MRONJ were included (Table 8): 19 case reports and case series, seven retrospective studies, four prospective studies, and two clinical trials.

**TABLE 8:** L‐PRF in the management of MRONJ: Studies included.

Study type	Number	References
Case report and case series	19	Bilimoria et al.^40^ Bouland et al.^41^ Cortese et al.^42^ Fernando de Almeida Barros Mourão et al.^43^ de Castro et al.^44^ Esen and Akkulah^45^ Giudice et al.^46^ Gönen and Yılmaz Asan^47^ Hao et al.^48^ Inchingolo et al.^49^ Law et al.^50^ Maluf et al.^51^ Maluf et al.^52^ Moraes‐da‐Silva et al.^53^ Pardo‐Zamora et al.^54^ Saad and Saad^55^ Şahin et al.^56^ Soydan and Uckan^18^ Tsai et al.^57^
Retrospective studies	7	Dincă et al.^58^ Özalp et al.^59^ Şahin et al.^60^ Szentpeteri et al.^61^ Tenore et al.^62^ Valente et al.^63^ Yalcin‐Ülker et al.^64^
Prospective studies	4	Gurav et al.^65^ Kim et al.^66^ Nørholt and Hartlev^67^ Zelinka et al.^68^
Case‐control studies/clinical trials	2	Giudice et al.^69^ Yüce et al.^70^

*Note*: Data related to the case reports (<5 patients included) are available in Tables S5 and S6.

All data concerning the studies are reported in Tables 9 and 10. A total of 306 patients with 320 lesions were recruited and underwent surgery with L‐PRF. Considering the clinical evaluations performed at the follow‐up visits (up to 108 months), the treatment outcomes included complete healing in 278 lesions (86.9%), partial healing in 20 lesions (6.2%), and no healing in 22 lesions (6.9%). No adverse effects were observed. It is important to emphasize that there was no uniformity in the description of the criteria for a partial or negative response to treatment (including recurrence).

**TABLE 9:** L‐PRF in the management of MRONJ: General features of the studies.

References	Patients number	Age (mean; range; years)	Gender (M/F)	Disease	Medication administered	Duration (mean, range or other)	Habits, comorbidities, other medications (if reported)
Bilimoria et al.^40^	5	65.6 (46–82)	2/3	Breast cancer 1 Multiple myeloma 3 Osteoporosis 1	*Cancer* Z 3 Z + D 1 *MBD* BP + Z	Cancer patients 5.75 years (range 2–10 years) MBD patients 11 y	Lupus, Sickle‐cell anemia 1
Fernando de Almeida Barros Mourão et al.^43^	11	67.7 ± 14.6 (38–84)	2/9	Osteoporosis	A	57.6 ± 14.7 m (range 36–84 m)	–
Dincă et al.^58^	10	59 + 15 (30–79)	4/6	Bowel cancer 1 Breast cancer 3 Kidney cancer 1 Multiple myeloma 2 Prostatic cancer 3	Z 7 I 3	–	–
Esen and Akkulah^45^	7	64.7 ± 9.3 (50–73)	1/6	Breast cancer 4 Prostate cancer 1 Osteoporosis 2	Z 4 I 2 R 1	–	–
Giudice et al.^69^	47 23 Surgery 24 Surgery + L‐PRF	74.7 + 6.5 (58–83) 73.9 + 7.4 (62–83) 75.5 + 5.6 (58–83)	23/24 9/14 14/10	Breast cancer 11 Kidney cancer 5 Lung cancer 3 Multiple myeloma 1 Osteoporosis 12 Prostatic cancer 15 Control Group Breast cancer 5 Kidney cancer 2 Lung cancer 2 Multiple myeloma 1 Osteoporosis 7 Prostatic cancer 5 L‐PRF Group Breast cancer 6 Kidney cancer 3 Lung cancer 1 Osteoporosis 5 Prostatic cancer 10	*Cancer* D 9 Z 26 *MBD* A 10 D 1 I 1 *Cancer* Z 12 D 4 *MBD* A 5 D 1 I 1 *Cancer* Z 14 D 5 *MBD* A 5	–	–
Gurav et al.^65^	15	61.3 ± 13.9	6/9	Breast cancer 6 Multiple myeloma 6 Renal cell carcinoma 1 Lung cancer 1 Prostate cancer 1	Z 12 Z + A + I 3	31 ± 13.9 doses	Sunitinib 1
Inchingolo et al.^49^	23	(52–73)	8/15	–	–	–	–
Kim et al.^66^	34	71 ± 13	0/34	Bone metastasis 2 Osteoporosis 32	*Cancer* Z 2 *MBD* A 19 R 8 P 4 Z1	78 m (range 21–92 m)	Chemotherapy 2 Diabetes 7 Obesity 4 Taking steroids 4 Renal failure 1
Nørholt and Hartlev^67^	15	68.5 (54–83)	4/11	Breast cancer 4 Kidney cancer 2 Multiple myeloma 1 Prostatic cancer 1 Osteoporosis 7	*Cancer* D 2 I 1 P 1 Z 4 *MBD* A 5 D 2	Cancer patients 34 m (range 15–73 m) MBD patients 126 m (range 48–240 m)	–
Özalp et al.^59^	13	72.4 ± 10.6 (54–84)	6/7	Prostate cancer 6 Breast cancer 3 Multiple myeloma 1 Osteoporosis 3	Z 11 A 1 I + Z + D 1	–	–
Yüce et al.^70^	28 14 L‐PRF 14 CTR	(65–81) 73.6 ± 5.1 73.6 ± 5.5	0/28	Osteoporosis	PRF A 13, R 1 CTR A 12, R 2	PRF 9.1 ± 2.2 years CTR 8.9 ± 2.0 years	PRF Hypertension 4 Diabetes 2 Autoimmune disease 1 CTR Hypertension 5 Diabetes 3
Şahin et al.^60^	21	68.0 ± 9.8 (49–85)	7/14	Breast cancer 14 Prostate cancer 3 Lung cancer 2 Kidney cancer 1 Multiple myeloma 1	Z	64.8 ± 21.5 m (range 39–96 m)	Smoking 6
Szentpeteri et al.^61^	28	68.4	11/17	Breast cancer 9 Prostate cancer 8 Multiple myeloma 4 Other cancer 4 Osteoporosis 3	IV BPs 20 Oral BPs 5 N/A 3	–	Chemotherapy 20 Hormone therapy 12
Tenore et al.^62^	13	72.2 ± 8.0 (58–82)	5/8	Breast cancer 2 Prostate cancer 3 Lung cancer 1 Bladder cancer 1 Multiple myeloma 3 Osteoporosis 3	*Cancer* Z 5 D 4 Z + D 1 *MBD* A 2 Z + A 1	Cancer patients 36.8 ± 27.4 m (range 10–97 m) MBD patients 42 ± 31.2 (range 6–60 m)	Chemotherapy 10 Corticosteroids 4 Diabetes 1 Smoking 6
Valente et al.^63^	14	64 (56–71)	6/9	Breast cancer 2 Melanoma 1 Multiple myeloma 1 Prostatic cancer 3 Osteoporosis 8	*Cancer* D 2 I 1 Z 4 *MBD* A 3 I 2 Z 2 D 1	2–6 years	Arthritis 1 Atrial fibrillation 3 Cardiovascular disease 13 Diabetes 4 Gastritis 4 Hypercholesterolemia 5 Hypertension 12 IMA 2 IRC 1 M. Parkinson 1 Smoking 1
Yalcin‐Ülker et al.^64^	19	63.8 ± 8.4	5/14	Breast cancer 12 Prostate cancer 2 Laryngeal cancer 1 Lung cancer 1 Parathyroid cancer 1 Multiple myeloma 1 Osteoporosis 1	Z 16 D 2 I 1	60.2 m (range 25–124 m)	Corticosteroid 3 Diabetes 1 Hypertension 1 Smoking 1
Zelinka et al.^68^	40	69 (37–85)	16/24	Prostate cancer 14 Breast cancer 13 (1 with B lymphoma) Lung cancer 3 Renal cancer 2 Multiple myeloma 1 Pancreatic cancer 1 Osteoporosis 6	D 17 BPs 10 D + BPs 17	52.8 m (range 4–144 m) Cancer patients 51.5 m (range 4–144 m) MBD patients 60.2 m (range 31–109 m)	Chemotherapy 11 Chemotherapy + corticosteroids 9 Corticosteroids 5 Diabetes 12 Smoking 2 Diabetes + smoking 2

*Note*: Zoledronic acid treatment for cancer patients usually involves administration of 4 mg every 4 weeks. Zoledronic acid treatment for MBD patients usually involves administration of 5 mg once a year. Denosumab treatment for cancer patients usually involves administration of 120 mg every 4 weeks. Denosumab treatment for MBD patients usually involves administration of 60 mg every 6 months.

Abbreviations: A, alendronic acid; B, bevacizumab; BP, bisphosphonate not specified; C, clodronic acid; CR, case reported; D, denosumab; I, ibandronic acid; MBD, metabolic bone disease; n.s, not specified; P, pamidronic acid; R, risedronic acid; S, sunitinib; Z, zoledronic acid.

**TABLE 10:** L‐PRF in the management of MRONJ: Specific features and outcomes of the studies.

References	Number of lesions	Site	Staging AAOMS (specified if different)	Type of intervention	Antibiotic therapy	Follow‐up	Outcome
Bilimoria et al.^40^	6	Mandible 4 Maxilla 2	Stage 2	Surgical debridement with piezoelectric device + L‐PRF	Amoxicillin 500 mg + metronidazole 400 mg × 3/day for 7 days	12 m	5 Complete healing 1 Partial healing
Fernando de Almeida Barros Mourão et al. 2020	11	Mandible 7 Maxilla 4	Stage 2	Sequestrectomy + L‐PRF	Amox/clav 2 g/day for 10 days (starting the day before surgery)	23.5 ± 8.7 m (range 12–36 m)	Complete healing
Dinca et al.^58^	10	Mandible 7 Maxilla 3	Stage 2	Surgical debridement and sequestrectomy + L‐PRF	Amox/clav 1 g × 4/day for 10 days	1 m	Complete healing
Esen and Akkulah^45^	7	Maxilla	Stage 3	Surgical debridement and sequestrectomy + L‐PRF + BFP (OAF closure)	Amox/clav 2 g/day Ornidazole 1 g/day for 2 weeks pre‐op Amox/clav 2 g/day for 5 days post‐op	14.6 m (range 12–18 m)	Complete healing
Giudice et al.^69^	61 28 Surgery 33 Surgery + L‐PRF	Mandible 49 Maxilla 12 Mandible 22 Maxilla 6 Mandible 27 Maxilla 6	27 Stage 2 20 Stage 3 13 Stage 2 10 Stage 3 14 Stage 2 10 Stage 3	Bone curettage Bone curettage + L‐PRF	Amoxicillin 1 g × 2/day + metronidazole 250 mg × 3/day or clindamycin 600 mg × 3/day Start 3 days before surgery, for 10 days	T1: 1 m T2: 6 m T3: 12 m	*Surgery* 26 Complete healing 2 No healing *Surgery + L‐PRF* 32 Complete healing 1 No healing
Gurav et al.^65^	16	–	10 Stage 2 6 Stage 3	Surgical debridement with piezoelectric device + L‐PRF	Amox/clav 500 + 125 mg	4 m	13 Complete healing 3 No healing
Inchingolo et al.^49^	23	–	–	Surgical debridement and sequestrectomy with piezoelectric device + L‐PRF	Amox/Clav 1 g × 2/day start 1 h before surgery, for 8 days	1 m	Complete healing
Kim et al.^66^	34	Mandible 27 Maxilla 7	7 Stage 1 21 Stage 2 6 Stage 3	Surgical debridement and sequestrectomy + L‐PRF	Third‐generation cephalosporin i.v. 1 g × 2/day	1, 4 m	26 Complete healing 6 Partial healing 2 No healing
Nørholt and Hartlev^67^	17	Mandible 13 Maxilla 4	13 Stage 2 4 Stage 3	Bone resection + L‐PRF	Penicillin 2 MIU + metronidazole 1 g preoperatively Penicillin 1 MIU × 4/day for 4 weeks Metronidazole 500 mg × 2/day for 5 days Clindamycin 600 mg × 3/day in case of allergy to penicillin	12 m (range 7–20 m)	15 Complete healing 2 No healing
Ozalp et al.^59^	13	Mandible 11 Maxilla 2	–	Surgical debridement + L‐PRF	Amox/clav 1 g 2×/day or clindamycin 150 mg 4×/day starting 2 days pre‐op, 7 days post‐op	20.1 ± 18.3 m	11 Complete healing (2 patient required re‐intervention) 2 Partial healing
Ozden Yuce et al.^70^	28 14 L‐PRF 14 CTR	PRF Mandible 9, Maxilla 5 CTR Mandible 10, Maxilla 4	PRF 8 Stage 2 6 Stage 3 CTR 7 Stage 2 7 Stage 3	PRF Surgical debridement and sequestrectomy + L‐PRF CTR Surgical debridement and sequestrectomy	Amox/clav 2 g/day 2 weeks pre‐op	6 m	PRF 11 Complete healing 3 No healing CTR 8 Complete healing 6 No healing *p* > 0.05
Sahin et al.^60^	21	Mandible 13 Maxilla 8	15 Stage 2 6 Stage 3	Surgical debridement and sequestrectomy + L‐PRF	Amox/clav 1 g Metronidazole 500 mg 1 week pre‐op, 2 weeks post‐op	18.04 ± 2.14 m	Complete healing (2 patients with delayed healing)
Szentpeteri et al.^61^	28	Mandible 17 Maxilla 8 Both jaws 3	21 Stage 2 7 Stage 3	Surgical debridement and sequestrectomy + L‐PRF	Amox/clav 1 g 2×/day or clindamycin 300 mg 4×/day starting 3 days pre‐op, 10 days post‐op	1 year	18 Complete healing 5 Partial healing (recurrence) 5 No healing (insufficient healing)
Tenore et al.^62^	13	Mandible 9 Maxilla 4	3 Stage 1 10 Stage 2	Surgical debridement and sequestrectomy + L‐PRF + PBM	Amox/clav 1 g 2×/day Metronidazole 250 mg 2×/day 3 days pre‐op, 7 days post‐op	6 m	Complete healing
Valente et al.^63^	14	Mandible 8 Maxilla 6	1 Stage 0 4 Stage 1 9 Stage 2 1 Stage 3	Surgical debridement and sequestrectomy + L‐PRF	Amoxicillin 2–3 g/day Clindamycin 900–1200 mg/day Ciprofloxacin 500 mg/day for 3–9 weeks	42.2 m (range 6–74 m)	10 Complete healing 4 No healing
Yalcin‐Ulker et al.^64^	20	Mandible 17 Maxilla 3	Stage 2 Stage 3	Surgical debridement and sequestrectomy + L‐PRF	Penicillin 1 200 000 IU/Clindamycin 600 mg pre‐op Amox/clav 1 g 2×/day for 1 week	27.9 ± 9.2 m (range 18–54 m)	18 Complete healing (2 with re‐intervention) 1 Partial healing 1 No healing
Zelinka et al.^68^	40	Mandible 25 Maxilla 15	1 Stage 0 3 Stage 1 21 Stage 2 15 Stage 3	Surgical debridement and sequestrectomy + L‐PRF	Amox/clav 1 g or clindamycin 300 mg 2 days pre‐op Intravenous antibiotic treatment 1 week Oral antibiotic treatment 1 week	1 year	34 Complete healing 5 Partial healing 1 No healing

Abbreviations: Amox/clav, amoxicillin + clavulanate; BFP, Buccal fat pad; GA, under general anesthesia; HBO, hyperbaric oxygen therapy; i.m., intramuscular administration; i.v., intravenous administration; PBM, photobiomodulation.

### 6.1 Comparison between treatments with/without L‐PRF

Among the articles included Giudice et al., and Yuce et al. designed their studies as clinical trials with two groups in which patients with MRONJ underwent surgical treatment with and without L‐PRF application.^69^, ^70^

Giudice et al. evaluated the effect of L‐PRF application after bone surgery in MRONJ patients (24 patients, 33 lesions) in terms of quality of life and mucosal healing and compared the results to those in patients who underwent bone surgery alone (23 patients, 28 lesions). Long‐term evaluation showed no statistical differences between the L‐PRF and non‐L‐PRF groups in terms of the absence of infection and mucosal healing; however, short‐term follow‐up showed significant improvement in terms of quality of life in favor of the L‐PRF group.^69^

Yuce et al. evaluated the effects of L‐PRF in 28 patients diagnosed with MRONJ. In the L‐PRF group (14 patients, 14 lesions), each patient was treated with L‐PRF after removing the necrotic bone before suturing, instead of surgical therapy alone as in the control group (14 patients, 14 lesions). At the 6 months follow‐up visit, complete healing was achieved in 19 of the 28 patients, with no significant differences between the groups. Among the nine patients in whom complete healing was not achieved, three were treated with L‐PRF (two with bone exposure without infection, one with recurrent infection) and six were treated with a traditional surgical protocol (three with bone exposure without infection, three with recurrent infection).^70^

Considering the aforementioned studies, L‐PRF application after surgery may increase healing in patients with MRONJ.

## 7 SYSTEMATIC REVIEWS

Six systematic reviews on the application of APCs as adjuvant treatment in the surgical management of MRONJ were included. Two studies focused only on L‐PRF application after the surgical treatment of MRONJ,^71^, ^72^ whereas the other four studies included articles related to PRP, PRGF, and L‐PRF as adjuvants after the surgical treatment of MRONJ.^13^, ^14^, ^15^, ^73^ The main findings of the six systematic reviews are presented in Table 11. In general, despite the success rate of APCs application as an adjuvant therapy after surgical treatment of MRONJ lesions, there are methodological limitations that reduce the scientific evidence to support its use.

**TABLE 11:** Systematic reviews on APCs application after surgical treatment of MRONJ.

References	APCs	Number of studies included	Meta‐analysis	Conclusions
Bracher et al.^71^	L‐PRF	16	No	The available evidence supporting the effectiveness of L‐PRF application in individuals with MRONJ is currently limited. This limitation is primarily attributed to variations in treatment definitions and administration methods, as well as small and sometimes non‐consecutive patient samples, along with the absence of randomized control groups. While the use of L‐PRF in MRONJ appears to be safe, the existing data only provides weak indications of potential benefits within the reviewed patient cohorts. Some observational reports and one randomized controlled trial have suggested improvements in early recovery. Consequently, further research is essential before any definitive recommendations regarding the role of L‐PRF in the management of MRONJ can be made
Del Fabbro et al.^13^	PRP, PRGF, L‐PRF	14	Yes (4 studies included – only PRP)	The findings of this review, drawn from studies with a low level of evidence, indicate that incorporating platelet concentrates as a supplement to oral surgery procedures might offer a positive impact in preventing the postsurgical recurrence of MRONJ in individuals undergoing bisphosphonate therapy
Escobedo et al.^73^	PRP, PRGF, L‐PRF	25	No	While the success rate following the implementation of adjunctive treatment with APCs for established MRONJs is notably high, this systematic review of the literature reveals significant methodological limitations that diminish the scientific evidence supporting its use. The articles included in the review exhibit substantial variations in study types, patient characteristics, and diverse medical and surgical protocols. Consequently, there is currently insufficient scientific data to robustly endorse the use of APCs in treating established MRONJs
Fortunato et al.^15^	PRP, PRGF, L‐PRF	35	No	The use of APCs holds promise in the treatment of MRONJ due to their localized immunomodulatory properties and potential facilitation of angiogenesis and tissue healing through platelet factors. However, given the constraints of this systematic review (such as the absence of studies eligible for meta‐analysis, a scarcity of randomized trials, the absence of multicentric studies, variations in the definition of treatment success, small sample sizes, various drug administrations, and differing protocols), findings do not provide sufficient evidence to establish its effectiveness
Lopez‐Jornet et al.^14^	PRP, PRGF, L‐PRF	8	No	There is a lack of scientific data that adequately supports any specific treatment protocol for the management of MRONJ, including the combination of APCs with surgical debridement
Muñoz‐Salgado et al.^72^	L‐PRF	10	Yes	The application of L‐PRF, either as a standalone treatment or in conjunction with other therapeutic approaches after MRONJ surgical treatment, resulted in a remarkable 94.3% achievement in complete lesion resolution. When compared to studies evaluating the efficacy of other therapies, the use of L‐PRF concurrently demonstrated higher rates of pathology resolution

## 8 DISCUSSION

The goal of MRONJ treatment is to simultaneously improve the patient's quality of life and manage bone infections, by preventing lesion extension at the same time.^74^ The type and duration of drug administration are among the factors that can influence the results of surgical treatment of MRONJ lesions. Additionally, patient health status should be considered, as all these variables could impact tissue healing.^10^

According to AAOMS 2014 Position Paper, the first choice in the management of MRONJ is conservative treatment with antibiotics and local antiseptics, suggesting surgical therapy in case of no response to medical therapy, in case of stage I or II, or in the management of stage III MRONJ.^75^

In the AAOMS 2022 Position Paper, Ruggiero et al. stated that despite conservative treatment continuing to be a treatment option for MRONJ, surgical treatment is increasingly being reported as a feasible option with high success rates for all stages of the disease. The choice between non‐surgical and surgical therapy should be a shared decision between the surgeon and the patient.^1^

The response to MRONJ treatment is influenced by several factors, including the type of drug (bisphosphonates or denosumab), dose of drug therapy according to the patient's primary disease (metabolic or malignant), and the combination of different drugs, including immune modulators or antiangiogenic medications.^1^ A group of researchers suggested that there is controversy in the AAOMS position paper regarding the definition, risk factors, classification, and management of MRONJ.^76^ Several authors have suggested that surgical management of MRONJ is indicated in the earlier stages of MRONJ to limit the spread of the infection and for bone removal.^77^, ^78^

The variables characterizing patients with MRONJ affect the interpretation of clinical outcomes related to the management of this disease. Treatment success can be defined as the complete resolution of a disease or, in some cases, as an improvement in the pathological state.^14^

The definition of resection margins for the complete removal of necrotic bone is also crucial for the success of the surgical management of MRONJ.^79^

The use of APCs as adjuvants in oral and periodontal surgeries is an important research topic. During the last 20 years, several authors have reported that APCs accelerate the healing of soft and hard tissues; however, their effectiveness in tissue regeneration remains controversial.^80^

Several protocols have been developed to obtain APCs; however, each product has a different potential use and biology.^81^ Epidermal growth factor (EGF), basic fibroblast growth factor (bFGF), insulin‐like growth factor‐1 (IGF‐I), platelet‐derived growth factor (PDGF), transforming growth factor β (TGF‐β), and vascular endothelial growth factor (VEGF) are GFs released by platelets and, consequently, APCs.^82^ These GFs have been shown to have chemotactic properties for various cell types, creating tissue microenvironments, and directly influencing the differentiation and proliferation of progenitor cells.^83^ PDGF enhances angiogenesis and osteogenesis in rats with induced bisphosphonate‐related osteonecrosis of the jaw with therapeutic effects.^84^

Miron et al. proposed to report in the methods section of studies related to L‐PRF all the data related to the centrifugation process including the centrifuge, the tubes, centrifugation speed (RCF) and time.^85^ Patient age, sex, and general health conditions, as well as technical parameters, can influence the fibrin network, antimicrobial efficacy, platelet count, and GF release.^86^, ^87^ In addition, the resting and compression times after centrifugation could affect the characteristics of APC membranes and their effectiveness.^88^ The polymerization modalities of the APCs seem to affect the release of GFs: PRP and PRGF polymerization are chemically induced, leading to an uncontrolled and short‐term release of GFs, whereas L‐PRF polymerizes slowly during centrifugation, resulting in a slow release of GFs that appear to persist for at least 14 days.^89^ However, no studies have compared the efficacy of PRP, PRGF, and L‐PRF in the treatment of MRONJ.

The results of this review showed favorable healing rates for MRONJ lesions managed with APCs after surgical treatment. However, a significant bias arose from the undefined definition of “healing” in almost all examined articles. The variable follow‐up periods considered in the studies included in this review made it challenging to compare the results. Only a few authors have examined the effects of platelet concentrates during short‐term follow‐up.^66^, ^69^

When comparing all the articles included in this review, there were other biases related to the study type, patient characteristics, and variations in medical and surgical protocols. These factors can potentially confound the evaluation of treatment outcomes. Some authors have used rotary instruments (burs), others have employed piezoelectric devices or lasers to remove the necrotic bone, while in a few cases, the tools used are not specified.^35^, ^40^, ^62^

Typical confounding factors include the type of jaw involved, location of MRONJ within the jaw, drug intake, comorbidities, age, and patient habits (smoking and alcohol intake). Garzino Demo et al. observed that their retrospective study lacked homogeneity and realism when combining data from oncological and non‐oncological patients with MRONJ. They suggested evaluating these groups as two distinct cohorts, owing to the potential influence of high‐dose or low‐dose BMA administration on the outcomes of surgical treatment.^90^ In a retrospective study, Kim et al. reported that patients taking glucocorticoids and those with diabetes mellitus have a higher risk of refractory healing after surgical treatment for MRONJ.^91^ Giudice et al. reported that patients under 70 years of age achieved better mucosal healing and MRONJ downstaging after surgical treatment than older patients.^78^

Some patients underwent surgery while still on a BMA or ACD, whereas others underwent surgery after a drug holiday. Although there is no consensus regarding the effectiveness of drug holidays on MRONJ lesions, it should be considered a potentially confounding factor in relation to treatment outcomes.^92^, ^93^

Another confounding factor affecting treatment outcomes, that is the extent of the necrotic bone lesions, was not considered in almost all studies. Zelinka et al. reported that in cases where complete healing was not achieved after surgical treatment, the initial defect size was significantly larger.^68^

The analysis of the results of the systematic literature reviews included in this study highlighted methodological limitations, both in the individual studies examined and in the meta‐analyses conducted by some authors.^13^, ^72^

Despite the obvious limitations of the scientific articles examined, several authors have reported promising results for the treatment of MRONJ using APCs as an adjunct to surgical treatment.^71^, ^72^ The use of APCs in the surgical treatment of MRONJ is supported by their ability to stabilize clots and improve soft tissue healing, as demonstrated in other oral surgical procedures.^94^ Furthermore, APCs act as a barrier to prevent contact between the oral mucosa and the bone, which can be useful in avoiding direct toxicity to soft tissues caused by bisphosphonates released from the bone after surgery.^67^, ^95^

Potential future uses of APCs in the management of MRONJ involve the possibility of combining drugs, such as antibiotics, with platelet concentrate to ensure controlled drug release at the site involved.^96^, ^97^, ^98^

In the most recent Cochrane review of interventions for MRONJ management, Beth‐Tasdogan et al. included only eight RCTs that compared different regimens for treating MRONJ. There is insufficient evidence to claim or refute the benefits of any of the tested interventions (hyperbaric oxygen therapy, fluorescence‐guided bone surgery, GFs application, and teriparatide administration) for the treatment of MRONJ. The small sample size may have contributed to the lack of measurable effects. Moreover, the methodological constraints of the trials were associated with a high risk of bias, contributing to uncertainty regarding the estimates of the effect.^19^

Future RCTs should address important practice‐related research questions related to the surgical treatment of MRONJ, including the evaluation of add‐on effects for adjunct treatments such as APCs application.

Beth‐Tasdogan et al. suggested a blinding of outcome assessors (data collectors), which is crucial to ensure unbiased outcome assessment, because blinding of participants and oral surgeons may not be possible because of the nature of most interventions. One important limitation of the existing RCTs is the small sample size of patients with MRONJ recruited in a single center, considering the rare event rates of MRONJ.^19^

Designing clinical trials involving patients with MRONJ presents several challenges and ethical considerations, particularly those concerning informed consent and the complexities of conducting research in vulnerable populations. It requires careful consideration of ethical principles such as respect for autonomy, beneficence, and justice, as well as practical strategies to address the unique challenges posed by this patient population.^99^, ^100^, ^101^ Researchers must prioritize patient safety and well‐being while advancing scientific knowledge and innovation in the treatment of MRONJ.

Future RCTs should follow a multicenter design and recruit an appropriate number of patients to draw meaningful conclusions regarding wound healing and PROMs. Given the limited number of available randomized controlled trials, further systematic literature reviews (with or without meta‐analyses) are not strictly necessary.

## 9 CONCLUSION

APCs may be helpful in the treatment of MRONJ because of their ability to promote angiogenesis, tissue healing and immunomodulation. However, considering the limitations of the studies included in this review (lack of a consistent or standardized definition of treatment success, heterogeneous drug administration, different surgical protocols, small sample sizes, only two randomized studies, and no multicenter studies), further multicenter RCTs with defined treatment criteria are needed to establish the extent to which APCs use could improve the tissue healing and quality of life in patients with MRONJ requiring surgical treatment.

## CONFLICT OF INTEREST STATEMENT

The authors declare that they have no competing interests related to this study.

## PATIENT CONSENT STATEMENT

Informed consent for image publication was obtained from the patient.

## Supporting information


Table S1.
Table S2.
Table S3.
Table S4.
Table S5.
Table S6.

## Data Availability

The data that support the findings of this study are available from the corresponding author upon reasonable request.

## References

[1] Ruggiero SL , Dodson TB , Aghaloo T , Carlson ER , Ward BB , Kademani D . American Association of Oral and Maxillofacial Surgeons' position paper on medication‐related osteonecrosis of the jaws—2022 update. J Oral Maxillofac Surg. 2022;80(5):920‐943. 35300956 10.1016/j.joms.2022.02.008

[2] King R , Tanna N , Patel V . Medication‐related osteonecrosis of the jaw unrelated to bisphosphonates and denosumab—a review. Oral Surg Oral Med Oral Pathol Oral Radiol. 2019;127(4):289‐299. 30713092 10.1016/j.oooo.2018.11.012

[3] Eguia A , Bagán‐Debón L , Cardona F . Review and update on drugs related to the development of osteonecrosis of the jaw. Med Oral Patol Oral Cir Bucal. 2020;25:e71‐e83. 31880288 10.4317/medoral.23191 PMC6982985

[4] Teoh L , Moses G , Nguyen AP , McCullough MJ . Medication‐related osteonecrosis of the jaw: analysing the range of implicated drugs from the Australian database of adverse event notifications. Br J Clin Pharmacol. 2021;87(7):2767‐2776. 33245790 10.1111/bcp.14681

[5] Campisi G , Alberto B , Fusco V , Società Italiana di Chirurgia Maxillo‐Facciale , Società di Patologia e Medicina Orale . Raccomandazioni clinico‐terapeutiche sull'osteonecrosi delle ossa mascellari (ONJ) farmaco‐relata e sua prevenzione. New Digital Press; 2020.

[6] Bennardo F , Buffone C , Giudice A . New therapeutic opportunities for COVID‐19 patients with tocilizumab: possible correlation of interleukin‐6 receptor inhibitors with osteonecrosis of the jaws. Oral Oncol. 2020;106:104659. 32209313 10.1016/j.oraloncology.2020.104659 PMC7270501

[7] Aghaloo T , Hazboun R , Tetradis S . Pathophysiology of osteonecrosis of the jaws. Oral Maxillofac Surg Clin North Am. 2015;27(4):489‐496. 26412796 10.1016/j.coms.2015.06.001 PMC4908822

[8] Chang J , Hakam AE , McCauley LK . Current understanding of the pathophysiology of osteonecrosis of the jaw. Curr Osteoporos Rep. 2018;16(5):584‐595. 30155844 10.1007/s11914-018-0474-4

[9] Diniz‐Freitas M , Limeres J . Prevention of medication‐related osteonecrosis of the jaws secondary to tooth extractions. A systematic review. Med Oral Patol Oral Cir Bucal. 2016;21:e250‐e259. 26827065 10.4317/medoral.20963 PMC4788807

[10] Yuan A , Munz A , Reinert S , Hoefert S . Gingival fibroblasts and medication‐related osteonecrosis of the jaw: results by real‐time and wound healing in vitro assays. J Craniomaxillofac Surg. 2019;47(9):1464‐1474. 31327558 10.1016/j.jcms.2019.06.004

[11] Di Fede O , Panzarella V , Mauceri R , et al. The dental management of patients at risk of medication‐related osteonecrosis of the jaw: new paradigm of primary prevention. Biomed Res Int. 2018;2018:1‐10. 10.1155/2018/2684924 PMC6164200 30306086

[12] Fortunato L , Barone S , Bennardo F , Giudice A . Management of facial pyoderma gangrenosum using platelet‐rich fibrin: a technical report. J Oral Maxillofac Surg. 2018;76(7):1460‐1463. 29425752 10.1016/j.joms.2018.01.012

[13] Del Fabbro M , Gallesio G , Mozzati M . Autologous platelet concentrates for bisphosphonate‐related osteonecrosis of the jaw treatment and prevention. A systematic review of the literature. Eur J Cancer. 2015;51(1):62‐74. 25466505 10.1016/j.ejca.2014.10.015

[14] Lopez‐Jornet P , Sanchez Perez A , Amaral Mendes R , Tobias A . Medication‐related osteonecrosis of the jaw: is autologous platelet concentrate application effective for prevention and treatment? A systematic review. J Craniomaxillofac Surg. 2016;44(8):1067‐1072. 27318752 10.1016/j.jcms.2016.05.004

[15] Fortunato L , Bennardo F , Buffone C , Giudice A . Is the application of platelet concentrates effective in the prevention and treatment of medication‐related osteonecrosis of the jaw? A systematic review. J Craniomaxillofac Surg. 2020;48(3):268‐285. 32063481 10.1016/j.jcms.2020.01.014

[16] Curi MM , Saraceni Issa Cossolin G , Koga DH , et al. Treatment of avascular osteonecrosis of the mandible in cancer patients with a history of bisphosphonate therapy by combining bone resection and autologous platelet‐rich plasma: report of 3 cases. J Oral Maxillofac Surg. 2007;65(2):349‐355. 17236949 10.1016/j.joms.2005.12.051

[17] Mozzati M , Gallesio G , Arata V , Pol R , Scoletta M . Platelet‐rich therapies in the treatment of intravenous bisphosphonate‐related osteonecrosis of the jaw: a report of 32 cases. Oral Oncol. 2012;48(5):469‐474. 22265335 10.1016/j.oraloncology.2011.12.004

[18] Soydan SS , Uckan S . Management of bisphosphonate‐related osteonecrosis of the jaw with a platelet‐rich fibrin membrane: technical report. J Oral Maxillofac Surg. 2014;72(2):322‐326. 24075235 10.1016/j.joms.2013.07.027

[19] Beth‐Tasdogan NH , Mayer B , Hussein H , Zolk O , Peter JU . Interventions for managing medication‐related osteonecrosis of the jaw. Cochrane Database Syst Rev. 2022;7(7):CD012432. 35866376 10.1002/14651858.CD012432.pub3 PMC9309005

[20] Hayashida S , Soutome S , Yanamoto S , et al. Evaluation of the treatment strategies for medication‐related osteonecrosis of the jaws (MRONJ) and the factors affecting treatment outcome: a multicenter retrospective study with propensity score matching analysis. J Bone Miner Res. 2017;32(10):2022‐2029. 28585700 10.1002/jbmr.3191

[21] Ruggiero SL , Kohn N . Disease stage and mode of therapy are important determinants of treatment outcomes for medication‐related osteonecrosis of the jaw. J Oral Maxillofac Surg. 2015;73(12):S94‐S100. 26608159 10.1016/j.joms.2015.09.024

[22] Adornato MC , Morcos I , Rozanski J . The treatment of bisphosphonate‐associated osteonecrosis of the jaws with bone resection and autologous platelet‐derived growth factors. J Am Dent Assoc. 2007;138(7):971‐977. 17606496 10.14219/jada.archive.2007.0294

[23] Antonini F , Pereira CCS , Parente EV , Azambuja FG . Management of osteonecrosis of the jaws in patients with history of bisphosphonates therapy. J Craniofac Surg. 2010;21(6):1962‐1966. 21119470 10.1097/SCS.0b013e3181f4ee4e

[24] Bernardi S , Di Girolamo M , Necozione S , Continenza MA , Cutilli T . Antiresorptive drug‐related osteonecrosis of the jaws, literature review and 5 years of experience. Musculoskelet Surg. 2019;103(1):47‐53. 29948937 10.1007/s12306-018-0548-6

[25] Cetiner S , Sucak GT , Kahraman SA , et al. Osteonecrosis of the jaw in patients with multiple myeloma treated with zoledronic acid. J Bone Miner Metab. 2009;27(4):435‐443. 19240969 10.1007/s00774-009-0047-9

[26] Coviello V , Peluso F , Dehkhargani SZ , et al. Platelet‐rich plasma improves wound healing in multiple myeloma bisphosphonate‐associated osteonecrosis of the jaw patients. J Biol Regul Homeost Agents. 2012;26(1):151‐155. 22475108

[27] Curi MM , Cossolin GSI , Koga DH , et al. Bisphosphonate‐related osteonecrosis of the jaws – an initial case series report of treatment combining partial bone resection and autologous platelet‐rich plasma. J Oral Maxillofac Surg. 2011;69(9):2465‐2472. 21763050 10.1016/j.joms.2011.02.078

[28] Lee CYS , David T , Nishime M . Use of platelet‐rich plasma in the management of oral biphosphonate‐associated osteonecrosis of the jaw: a report of 2 cases. J Oral Implantol. 2007;33(6):371‐382. 18240798 10.1563/1548-1336(2007)33[371:UOPPIT]2.0.CO;2

[29] Merigo E , Cella L , Oppici A , et al. Combined approach to treat medication‐related osteonecrosis of the jaws. J Lasers Med Sci. 2018;9(2):92‐100. 30026893 10.15171/jlms.2018.19 PMC6046388

[30] Vairaktaris E , Vassiliou S , Avgoustidis D , Stathopoulos P , Toyoshima T , Yapijakis C . Bisphosphonate‐induced avascular osteonecrosis of the mandible associated with a common thrombophilic mutation in the prothrombin gene. J Oral Maxillofac Surg. 2009;67(9):2009‐2012. 19686941 10.1016/j.joms.2009.04.032

[31] Longo F , Guida A , Aversa C , et al. Platelet rich plasma in the treatment of bisphosphonate‐related osteonecrosis of the jaw: personal experience and review of the literature. Int J Dent. 2014;2014:298945. 25013411 10.1155/2014/298945 PMC4071853

[32] Martins MAT , Martins MD , Lascala CA , et al. Association of laser phototherapy with PRP improves healing of bisphosphonate‐related osteonecrosis of the jaws in cancer patients: a preliminary study. Oral Oncol. 2012;48(1):79‐84. 21940198 10.1016/j.oraloncology.2011.08.010

[33] Mathias Duarte LFS , dos Reis HB , Tucci R , Dib LL . Bisphosphonate‐related osteonecrosis of the jaws: analysis of a case series at a dental school. Spec Care Dentist. 2014;34(2):77‐83. 23875734 10.1111/scd.12023

[34] Bocanegra‐Pérez S , Vicente‐Barrero M , Knezevic M , et al. Use of platelet‐rich plasma in the treatment of bisphosphonate‐related osteonecrosis of the jaw. Int J Oral Maxillofac Surg. 2012;41(11):1410‐1415. 22647765 10.1016/j.ijom.2012.04.020

[35] Mauceri R , Panzarella V , Maniscalco L , et al. Conservative surgical treatment of bisphosphonate‐related osteonecrosis of the jaw with Er,Cr:YSGG laser and platelet‐rich plasma: a longitudinal study. Biomed Res Int. 2018;2018:3982540. 30211221 10.1155/2018/3982540 PMC6120338

[36] Anitua E , Begoña L , Orive G . Treatment of hemimandibular paresthesia in a patient with bisphosphonate‐related osteonecrosis of the jaw (BRONJ) by combining surgical resection and PRGF‐Endoret. Br J Oral Maxillofac Surg. 2013;51(8):e272‐e274. 23201057 10.1016/j.bjoms.2012.08.018

[37] Gil IG , Ponte BM , Mateo ST , García JJ . Treatment of bisphosphonate‐related osteonecrosis of the jaw with plasma rich in growth factors after dental implant surgery: a case report. J Oral Implantol. 2019;45(4):289‐296. 31207196 10.1563/aaid-joi-D-18-00254

[38] Pardiñas López S , Iocca O , Khouly I . Three‐dimensional bone evaluation after surgical treatment with plasma rich in growth factors of medication related osteonecrosis of the jaw (MRONJ): a report of 3 cases. Bone Rep. 2019;10:100208. 31193239 10.1016/j.bonr.2019.100208 PMC6522655

[39] Sánchez‐Gallego Albertos C , Pardo DC , de Vera JL , Viejo Llorente A , Cebrián Carretero JL . Medication related osteonecrosis of the jaws (MRONJ): factors related to recurrence after treatment with surgery and platelet rich plasma (PRP) placement. Med Oral Patol Oral Cir Bucal. 2021;26(6):e684‐e690. 34704981 10.4317/medoral.24007 PMC8601641

[40] Bilimoria R , Young H , Patel D , Kwok J . The role of piezoelectric surgery and platelet‐rich fibrin in treatment of ORN and MRONJ: a clinical case series. Oral Surg. 2018;11(2):136‐143.

[41] Bouland C , Meuleman N , Widelec J , et al. Case reports of medication‐related osteonecrosis of the jaw (MRONJ) treated with uncultured stromal vascular fraction and L‐PRF. J Stomatol Oral Maxillofac Surg. 2021;122(2):212‐218. 32540361 10.1016/j.jormas.2020.05.024

[42] Cortese A , Casarella A , Howard CM , Claudio PP . Epi‐mucosa fixation and autologous platelet‐rich fibrin treatment in medication‐related osteonecrosis of the jaw. Dent J. 2021;9(5):50. 10.3390/dj9050050 PMC8146740 33946237

[43] Fernando de Almeida Barros Mourão C , Calasans‐Maia MD , Del Fabbro M , et al. The use of platelet‐rich fibrin in the management of medication‐related osteonecrosis of the jaw: a case series. J Stomatol Oral Maxillofac Surg. 2020;121(1):84‐89. 30794883 10.1016/j.jormas.2019.02.011

[44] de Castro MS , Ribeiro NV , de Carli ML , Pereira AAC , Sperandio FF , Hanemann JAC . Photodynamically dealing with bisphosphonate‐related osteonecrosis of the jaw: successful case reports. Photodiagnosis Photodyn Ther. 2016;16:72‐75. 27585753 10.1016/j.pdpdt.2016.08.007

[45] Esen A , Akkulah S . Management of large oroantral fistulas caused by medication‐related osteonecrosis with the combined sequestrectomy, buccal fat pad flap and platelet‐rich fibrin. J Maxillofac Oral Surg. 2021;20(1):76‐82. 33584046 10.1007/s12663-019-01278-x PMC7855103

[46] Giudice A , Antonelli A , Muraca D , Fortunato L . Usefulness of advanced‐platelet rich fibrin (A‐PRF) and injectable‐platelet rich fibrin (i‐PRF) in the management of a massive medication‐related osteonecrosis of the jaw (MRONJ): a 5‐years follow‐up case report. Indian J Dent Res. 2020;31(5):813. 33433526 10.4103/ijdr.IJDR_689_19

[47] Gönen ZB , Yılmaz Asan C . Treatment of bisphosphonate‐related osteonecrosis of the jaw using platelet‐rich fibrin. Cranio. 2017;35(5):332‐336. 27363584 10.1080/08869634.2016.1203093

[48] Hao L , Tian Z , Li S , Yan K , Xue Y . Osteonecrosis of the jaw induced by bisphosphonates therapy in bone metastases patient: case report and literature review. Oral Oncol. 2022;128:105852. 35439709 10.1016/j.oraloncology.2022.105852

[49] Inchingolo F , Cantore S , Dipalma G , et al. Platelet rich fibrin in the management of medication‐related osteonecrosis of the jaw: a clinical and histopathological evaluation. J Biol Regul Homeost Agents. 2017;31(3):811‐816. 28958140

[50] Law B , Soh HY , Nabil S , Rajandram RK , Nazimi AJ , Ramli R . Autologous platelet‐rich fibrin (PRF) as an adjunct in the management of osteoradionecrosis and medication‐related osteonecrosis of jaws. Case series in a single centre. Appl Sci. 2021;11(8):3365.

[51] Maluf G , Pinho MC d , Cunha SR d B d , Santos PS d S , Fregnani ER . Surgery combined with LPRF in denosumab osteonecrosis of the jaw: case report. Braz Dent J. 2016;27(3):353‐358. 27224573 10.1590/0103-6440201600662

[52] Maluf G , Caldas RJ , Silva Santos PS . Use of leukocyte‐ and platelet‐rich fibrin in the treatment of medication‐related osteonecrosis of the jaws. J Oral Maxillofac Surg. 2018;76(1):88‐96. 28675810 10.1016/j.joms.2017.06.004

[53] Moraes‐da‐Silva A d F , Maluf G , Rubira‐Bullen IRF , Santos PS d S . Bone morphogenetic protein 2 plus leukocyte and platelet‐rich fibrin for the treatment of MRONJ. J Craniofac Surg. 2023;34(4):e338‐e341. 36217220 10.1097/SCS.0000000000009059

[54] Pardo‐Zamora G , Martínez Y , Moreno JA , Ortiz‐Ruíz AJ . Treatment of stage 2 medication‐induced osteonecrosis of the jaw: a case series. Int J Environ Res Public Health. 2021;18(3):1018. 33498884 10.3390/ijerph18031018 PMC7908594

[55] Saad D , Saad P . Report of a jaw osteonecrosis possibly caused by denosumab. Eur J Oral Implantol. 2017;10(2):213‐222. 28555210

[56] Şahin O , Odabaşi O , Ekmekcioğlu C . Ultrasonic piezoelectric bone surgery combined with leukocyte and platelet‐rich fibrin and pedicled buccal fat pad flap in denosumab‐related osteonecrosis of the jaw. J Craniofac Surg. 2019;30(5):e434‐e436. 31299805 10.1097/SCS.0000000000005472

[57] Tsai LL , Huang YF , Chang YC . Treatment of bisphosphonate‐related osteonecrosis of the jaw with platelet‐rich fibrin. J Formos Med Assoc. 2016;115(7):585‐586. 26596688 10.1016/j.jfma.2015.10.005

[58] Dincă O , Zurac S , Stăniceanu F , et al. Clinical and histopathological studies using fibrin‐rich plasma in the treatment of bisphosphonate‐related osteonecrosis of the jaw. Rom J Morphol Embryol. 2014;55(3):961‐964. 25329128

[59] Özalp Ö , Yıldırımyan N , Öztürk C , et al. Promising results of surgical management of advanced medication related osteonecrosis of the jaws using adjunctive leukocyte and platelet rich fibrin. BMC Oral Health. 2021;21(1):613. 34852823 10.1186/s12903-021-01965-7 PMC8638116

[60] Şahin O , Akan E , Tatar B , Ekmekcioğlu C , Ünal N , Odabaşı O . Combined approach to treatment of advanced stages of medication‐related osteonecrosis of the jaw patients. Braz J Otorhinolaryngol. 2022;88(4):613‐620. 34023243 10.1016/j.bjorl.2021.04.004 PMC9422660

[61] Szentpeteri S , Schmidt L , Restar L , Csaki G , Szabo G , Vaszilko M . The effect of platelet‐rich fibrin membrane in surgical therapy of medication‐related osteonecrosis of the jaw. J Oral Maxillofac Surg. 2020;78(5):738‐748. 31945309 10.1016/j.joms.2019.12.008

[62] Tenore G , Zimbalatti A , Rocchetti F , et al. Management of medication‐related osteonecrosis of the jaw (MRONJ) using leukocyte‐ and platelet‐rich fibrin (L‐PRF) and Photobiomodulation: a retrospective study. J Clin Med. 2020;9(11):3505. 33138266 10.3390/jcm9113505 PMC7693575

[63] Valente NA , Chatelain S , Alfonsi F , Mortellaro C , Barone A . Medication‐related osteonecrosis of the jaw: the use of leukocyte‐platelet‐rich fibrin as an adjunct in the treatment. J Craniofac Surg. 2019;30(4):1095‐1101. 30908443 10.1097/SCS.0000000000005475

[64] Yalcin‐Ülker GM , Duygu G , Tanan G , Cakir M , Meral DG . Use of leukocyte‐rich and platelet‐rich fibrin (L‐PRF) adjunct to surgical debridement in the treatment of stage 2 and 3 medication‐related osteonecrosis of the jaw. J Craniofac Surg. 2023;34(3):1039‐1044. 36627754 10.1097/SCS.0000000000009161

[65] Gurav S , Dholam KP , Singh GP . Treatment of refractory medicine related osteonecrosis of jaw with piezosurgical debridement and autologous platelet rich fibrin: feasibility study. J Craniofac Surg. 2022;33(3):e226‐e230. 34310422 10.1097/SCS.0000000000007981

[66] Kim JW , Kim SJ , Kim MR . Leucocyte‐rich and platelet‐rich fibrin for the treatment of bisphosphonate‐related osteonecrosis of the jaw: a prospective feasibility study. Br J Oral Maxillofac Surg. 2014;52(9):854‐859. 25138613 10.1016/j.bjoms.2014.07.256

[67] Nørholt SE , Hartlev J . Surgical treatment of osteonecrosis of the jaw with the use of platelet‐rich fibrin: a prospective study of 15 patients. Int J Oral Maxillofac Surg. 2016;45(10):1256‐1260. 27179556 10.1016/j.ijom.2016.04.010

[68] Zelinka J , Blahak J , Perina V , Pacasova R , Treglerova J , Bulik O . The use of platelet‐rich fibrin in the surgical treatment of medication‐related osteonecrosis of the jaw: 40 patients prospective study. Biomed Pap. 2021;165(3):322‐327. 10.5507/bp.2020.023 32597421

[69] Giudice A , Barone S , Giudice C , Bennardo F , Fortunato L . Can platelet‐rich fibrin improve healing after surgical treatment of medication‐related osteonecrosis of the jaw? A pilot study. Oral Surg Oral Med Oral Pathol Oral Radiol. 2018;126(5):390‐403. 30108028 10.1016/j.oooo.2018.06.007

[70] Yüce MO , Adalı E , Işık G . The effect of concentrated growth factor (CGF) in the surgical treatment of medication‐related osteonecrosis of the jaw (MRONJ) in osteoporosis patients: a randomized controlled study. Clin Oral Investig. 2021;25(7):4529‐4541. 10.1007/s00784-020-03766-8 33392802

[71] Bracher AI , Vig N , Burkhard JP , Schaller B , Schlittler F . The application of platelet rich fibrin in patients presenting with osteonecrosis of the jaw: a systematic literature review. Adv Oral Maxillofac Surg. 2021;2:100076.

[72] Muñoz‐Salgado A , Silva FF , Padín‐Iruegas ME , et al. Leukocyte and platelet rich fibrin in the management of medication‐related osteonecrosis of the jaw: a systematic review and meta‐analysis. Med Oral Patol Oral Cir Bucal. 2023;28(4):e317‐e329. 36641740 10.4317/medoral.25733 PMC10314351

[73] Escobedo MF , Junquera S , Gonzalez C , et al. Efficacy of complementary treatment with autologous platelet concentrates and/or mesenchymal stem cells in chemical osteonecrosis of the jaw. Systematic review of the literature. J Stomatol Oral Maxillofac Surg. 2022;123(1):51‐58. 33609789 10.1016/j.jormas.2021.01.015

[74] Allen MR , Ruggiero SL . A review of pharmaceutical agents and oral bone health: how osteonecrosis of the jaw has affected the field. Int J Oral Maxillofac Implants. 2014;29(1):e45‐e57. 24451887 10.11607/jomi.te41

[75] Ruggiero SL , Dodson TB , Fantasia J , et al. American Association of Oral and Maxillofacial Surgeons position paper on medication‐related osteonecrosis of the jaw—2014 update. J Oral Maxillofac Surg. 2014;72(10):1938‐1956. 25234529 10.1016/j.joms.2014.04.031

[76] Schiodt M , Otto S , Fedele S , et al. Workshop of European task force on medication‐related osteonecrosis of the jaw—current challenges. Oral Dis. 2019;25(7):1815‐1821. 31325201 10.1111/odi.13160

[77] Ristow O , Rückschloß T , Müller M , et al. Is the conservative non‐surgical management of medication‐related osteonecrosis of the jaw an appropriate treatment option for early stages? A long‐term single‐center cohort study. J Craniomaxillofac Surg. 2019;47(3):491‐499. 30642734 10.1016/j.jcms.2018.12.014

[78] Giudice A , Barone S , Diodati F , Antonelli A , Nocini R , Cristofaro MG . Can surgical management improve resolution of medication‐related osteonecrosis of the jaw at early stages? A prospective cohort study. J Oral Maxillofac Surg. 2020;78(11):1986‐1999. 32615096 10.1016/j.joms.2020.05.037

[79] Wehrhan F , Weber M , Neukam FW , Geppert CI , Kesting M , Preidl RHM . Fluorescence‐guided bone resection: a histological analysis in medication‐related osteonecrosis of the jaw. J Craniomaxillofac Surg. 2019;47(10):1600‐1607. 31387830 10.1016/j.jcms.2019.07.012

[80] Bennardo F , Bennardo L , Del Duca E , et al. Autologous platelet‐rich fibrin injections in the management of facial cutaneous sinus tracts secondary to medication‐related osteonecrosis of the jaw. Dermatol Ther. 2020;33(3):e13334. 32219975 10.1111/dth.13334

[81] Dohan Ehrenfest DM , Rasmusson L , Albrektsson T . Classification of platelet concentrates: from pure platelet‐rich plasma (P‐PRP) to leucocyte‐ and platelet‐rich fibrin (L‐PRF). Trends Biotechnol. 2009;27(3):158‐167. 19187989 10.1016/j.tibtech.2008.11.009

[82] Etulain J . Platelets in wound healing and regenerative medicine. Platelets. 2018;29(6):556‐568. 29442539 10.1080/09537104.2018.1430357

[83] Miron RJ , Fujioka‐Kobayashi M , Bishara M , Zhang Y , Hernandez M , Choukroun J . Platelet‐rich fibrin and soft tissue wound healing: a systematic review. Tissue Eng Part B Rev. 2017;23(1):83‐99. 27672729 10.1089/ten.TEB.2016.0233

[84] Gao S y , Lin R b , Huang S h , et al. PDGF‐BB exhibited therapeutic effects on rat model of bisphosphonate‐related osteonecrosis of the jaw by enhancing angiogenesis and osteogenesis. Bone. 2021;144:115117. 31676407 10.1016/j.bone.2019.115117

[85] Miron RJ , Pinto NR , Quirynen M , Ghanaati S . Standardization of relative centrifugal forces in studies related to platelet‐rich fibrin. J Periodontol. 2019;90(8):817‐820. 30730050 10.1002/JPER.18-0553

[86] Miron RJ , Dham A , Dham U , Zhang Y , Pikos MA , Sculean A . The effect of age, gender, and time between blood draw and start of centrifugation on the size outcomes of platelet‐rich fibrin (PRF) membranes. Clin Oral Investig. 2019;23(5):2179‐2185. 10.1007/s00784-018-2673-x 30280327

[87] Mamajiwala AS , Sethi KS , Raut CP , Karde PA , Mangle NM . Impact of different platelet‐rich fibrin (PRF) procurement methods on the platelet count, antimicrobial efficacy, and fibrin network pattern in different age groups: an in vitro study. Clin Oral Investig. 2020;24(5):1663‐1675. 10.1007/s00784-019-03022-8 31346783

[88] Wei Y , Cheng Y , Wang Y , Zhang X , Miron RJ , Zhang Y . The effect of resting and compression time post‐centrifugation on the characteristics of platelet rich fibrin (PRF) membranes. Clin Oral Investig. 2022;26(8):5281‐5288. 10.1007/s00784-022-04496-9 35451655

[89] He L , Lin Y , Hu X , Zhang Y , Wu H . A comparative study of platelet‐rich fibrin (PRF) and platelet‐rich plasma (PRP) on the effect of proliferation and differentiation of rat osteoblasts in vitro. Oral Surg Oral Med Oral Pathol Oral Radiol Endod. 2009;108(5):707‐713. 19836723 10.1016/j.tripleo.2009.06.044

[90] Garzino Demo P , Bojino A , Roccia F , Malandrino MC , Cocis S , Ramieri G . Different presentation and outcomes in the surgical treatment of advanced MRONJ in oncological and nononcological patients taking or not corticosteroid therapy. Biomed Res Int. 2021;2021:7855497. 38523862 10.1155/2021/7855497 PMC10960651

[91] Kim JY , Song HC , Jee HG . Refractory healing after surgical therapy of osteonecrosis of the jaw: associated risk factors in aged patients. Clin Interv Aging. 2019;14:797‐804. 31123397 10.2147/CIA.S200455 PMC6510385

[92] Ottesen C , Schiodt M , Gotfredsen K . Efficacy of a high‐dose antiresorptive drug holiday to reduce the risk of medication‐related osteonecrosis of the jaw (MRONJ): a systematic review. Heliyon. 2020;6(4):e03795. 32373730 10.1016/j.heliyon.2020.e03795 PMC7191576

[93] Morishita K , Soutome S , Otsuru M , et al. Relationship between drug holiday of the antiresorptive agents and surgical outcome of medication‐related osteonecrosis of the jaw in osteoporosis patients. Sci Rep. 2022;12(1):11545. 35799050 10.1038/s41598-022-15720-7 PMC9263140

[94] Brancaccio Y , Antonelli A , Barone S , Bennardo F , Fortunato L , Giudice A . Evaluation of local hemostatic efficacy after dental extractions in patients taking antiplatelet drugs: a randomized clinical trial. Clin Oral Investig. 2021;25(3):1159‐1167. 10.1007/s00784-020-03420-3 32613433

[95] Choukroun J , Diss A , Simonpieri A , et al. Platelet‐rich fibrin (PRF): a second‐generation platelet concentrate. Part IV: clinical effects on tissue healing. Oral Surg Oral Med Oral Pathol Oral Radiol Endod. 2006;101(3):e56‐e60. 16504852 10.1016/j.tripleo.2005.07.011

[96] Siawasch SAM , Andrade C , Castro AB , Teughels W , Temmerman A , Quirynen M . Impact of local and systemic antimicrobials on leukocyte‐ and platelet rich fibrin: an in vitro study. Sci Rep. 2022;12(1):2710. 35177676 10.1038/s41598-022-06473-4 PMC8854700

[97] Bennardo F , Gallelli L , Palleria C , et al. Can platelet‐rich fibrin act as a natural carrier for antibiotics delivery? A proof‐of‐concept study for oral surgical procedures. BMC Oral Health. 2023;23(1):134. 36894902 10.1186/s12903-023-02814-5 PMC9996939

[98] Stapf M , Straub A , Fischer M , Linz C , Hartmann S , Scherf‐Clavel O . A liquid chromatography‐tandem mass spectrometry method for the quantification of ampicillin/sulbactam and clindamycin in jawbone, plasma, and platelet‐rich fibrin: application to patients with osteonecrosis of the jaw. J Pharm Biomed Anal. 2023;224:115167. 36435082 10.1016/j.jpba.2022.115167

[99] Iacono T . Ethical challenges and complexities of including people with intellectual disability as participants in research. J Intellect Dev Disabil. 2006;31(3):173‐179. 16954097 10.1080/13668250600876392

[100] Sharkey S , Jones R , Smithson J , et al. Ethical practice in internet research involving vulnerable people: lessons from a self‐harm discussion forum study (SharpTalk). J Med Ethics. 2011;37(12):752‐758. 21947802 10.1136/medethics-2011-100080

[101] Macdonald ME , Muirhead V , Doughty J , Freeman R . Critically engaging vulnerability: rethinking oral health with vulnerabilized populations. Community Dent Oral Epidemiol. 2022;50(6):469‐475. 34751455 10.1111/cdoe.12703

