# Supplementary material for: Autologous platelet concentrates as adjuvant in the surgical management of medication‐related osteonecrosis of the jaw
Source: Periodontol 2000. 2024 Sep 30;97(1):287–307. doi: 10.1111/prd.12608 (PMC11808450; doi:10.1111/prd.12608)
Supplement: Supplementary file 1 — Table S1. Table S2. Table S3. Table S4. Table S5. Table S6. [file PRD-97-287-s001.docx]

Supplementary Table 1 – PRP in the management of MRONJ: general features of case reports

| **Reference** | **Patients number** | **Age (mean; range; years)** | **Gender (M/F)** | **Disease** | **Medication administered** | **Duration (mean, range or other)** | **Habits, comorbidities, other medications (if reported)** |
| --- | --- | --- | --- | --- | --- | --- | --- |
| Antonini et al, 2010^23^ | 1 | 72 | 0/1 | Breast cancer | Z | 4 y | - |
| Bernardi et al, 2019^24^ | 1 | 68 | 0/1 | Osteoporosis | I | - | - |
| Cetiner et al, 2009^25^ | 1 | 68 | 1/0 | Multiple myeloma | Z | 7 m | - |
| Coviello et al, 2012^26^ | 3 Surgery + PRP | 76±7.5 (69-84) | 1/2 | Multiple myeloma | 3 Z | 8.5 y Z | - |
| Curi et al, 2007^16^ | 3 | 66.3±3.2 | 0/3 | Multiple myeloma 1  Breast cancer 2 | Z | 8.5 y | - |
| Lee et al, 2007^28^ | 2 | 80±5.6 (76-84) | 1/1 | Osteoporosis | A | 5-9 y | Chronic pulmonary obstructive disease 1  Hypertension 1  Pneumonia 1 |
| Vairaktaris et al, 2009^30^ | 1 | 72 | 0/1 | Breast cancer | P | 3 y | Prothrombin gene G21020A mutation (associated with thrombophilia) |

Legend

Z: zoledronic acid; P: pamidronic acid; I: ibandronic acid; C: clodronic acid; R: risedronic acid; D: denosumab; S: sunitinib; B: bevacizumab; A: alendronic acid; CR: case reported ;n.s.: not specified; N/A: not available; BP: bisphosphonate not specified; MBD: metabolic bone disease

Zoledronic acid treatment for cancer patients usually involves administration of 4 mg every 4 weeks

Zoledronic acid treatment for MBD patients usually involves administration of 5 mg once a year

Denosumab treatment for cancer patients usually involves administration of 120 mg every 4 weeks

Denosumab treatment for MBD patients usually involves administration of 60 mg every 6 months.

Supplementary Table 2 - PRP in the management of MRONJ: specific features and outcomes of case reports

| **Reference** | **Number of lesions** | **Site** | **Staging AAOMS (specified if different)** | **Type of intervention** | **Antibiotic therapy** | **Follow-up** | **Outcome** |
| --- | --- | --- | --- | --- | --- | --- | --- |
| Antonini et al, 2010^23^ | 1 | Maxilla | Stage 3 | Bone resection + HBO + PRP | Cephalexin 500 mg x 4/d for 10 d | 12 m | Complete healing |
| Bernardi et al, 2019^24^ | 1 | Mandible | Stage 3 | Surgical debridement and fracture reduction (GA) + PRP | - | 12 m | Complete healing |
| Cetiner et al, 2009^25^ | 1 | Mandible 1 | - | Bone resection + PRP | Amox/clav 1g x 2/d or Clindamycin 150–300 mg x 2–4/d | 6 m | Complete healing |
| Coviello et al, 2012^26^ | 4 | Mandible 3  Maxilla 1 | - | Surgical debridement and sequestrectomy + PRP | Amox/clav 1g x 2/d for 14 d | 3 m | Complete healing |
| Curi et al, 2007^16^ | 3 | Mandible  3 | - | Bone resection + PRP | Clindamycin 300 mg x 4/d for 14 days | 6 m | Complete healing 2 Partial healing 1 |
| Lee et al, 2007^28^ | 2 | Mandible 1  Maxilla 1 | - | Surgical debridement + PRP  Case 1: HBO | Penicillin i.v. for 3 m, then oral penicillin VK for 3 m | 7 m | Complete healing |
| Vairaktaris et al, 2009^30^ | 1 | Mandible | - | Bone resection + PRP | - | 4 m | Partial healing |

Legend

Amox/clav: amoxicillin + clavulanate

BFP: Buccal fat pad

GA: under general anesthesia

HBO: hyperbaric oxygen therapy

i.m.: intramuscular administration

i.v.: intravenous administration

N/A: not available

Supplementary Table 3 – PRGF in the management of MRONJ: general features of case reports

| **Reference** | **Patients number** | **Age (mean; range; years)** | **Gender (M/F)** | **Disease** | **Medication administered** | **Duration (mean, range or other)** | **Habits, comorbidities, other medications (if reported)** |
| --- | --- | --- | --- | --- | --- | --- | --- |
| Anitua et al, 2013^36^ | 1 | 50 | 0/1 | Cancer | Z | 3 y | - |
| Gil et al, 2019^37^ | 1 | 65 | 0/1 | Osteoporosis | A | 1 y | Epilepsy  Hypertension |
| Pardinas Lopez et al, 2019^38^ | 3 | 69.3 (61-80) | 0/3 | Breast cancer 1  Osteoporosis 2 | Cancer  Z 1  MBD  R 1  I 1 | Case 1 9y  Case 2 6y  Case 3 3y | Smoking 1  Radiotherapy, and Chemotherapy 1 |

Legend

Z: zoledronic acid; P: pamidronic acid; I: ibandronic acid; R: risedronic acid; D: denosumab; A: alendronic acid; N/A: not available; BP: bisphosphonate not specified; MBD: metabolic bone disease

Zoledronic acid treatment for cancer patients usually involves administration of 4 mg every 4 weeks

Zoledronic acid treatment for MBD patients usually involves administration of 5 mg once a year

Denosumab treatment for cancer patients usually involves administration of 120 mg every 4 weeks

Denosumab treatment for MBD patients usually involves administration of 60 mg every 6 months

Supplementary Table 4 – PRGF in the management of MRONJ: specific features and outcomes of case reports

| **Reference** | **Number of lesions** | **Site** | **Staging AAOMS (specified if different)** | **Type of intervention** | **Antibiotic therapy** | **Follow-up** | **Outcome** |
| --- | --- | --- | --- | --- | --- | --- | --- |
| Anitua et al, 2013^36^ | 1 | Mandible | - | Bone resection + PRGF | - | 12 m | Complete healing |
| Gil et al, 2019^37^ | 1 | Mandible | - | Surgical debridement and sequestrectomy + PRGF | Amox/clav 875/125 mg for 7 d | 3,6,12,24 m | Complete healing |
| Pardinas Lopez et al, 2019^38^ | 3 | Mandible | Stage 2 | Bone resection + PRGF | Amoxicillin 2g 1 hour before surgery  Amoxicillin 500 mg x 3/d for 10 d in Cases 2 and 3, for 21 d in Case 1 | 30 m | Complete healing |

Legend

Amox/clav: amoxicillin + clavulanate

N/A: not available

Supplementary Table 5 – L-PRF in the management of MRONJ: general features of case reports

| **Reference** | **Patients number** | **Age (mean; range; years)** | **Gender (M/F)** | **Disease** | **Medication administered** | **Duration (mean, range or other)** | **Habits, comorbidities, other medications (if reported)** |
| --- | --- | --- | --- | --- | --- | --- | --- |
| Bouland et al, 2021^41^ | 2 | 77  76 | 0/2 | Multiple myeloma  Osteoporosis | Z  Z | 1y  10y | Parkinson, breast cancer, hysterectomy, knee prosthesis |
| Cortese et al, 2021^42^ | 2 | 72  80 | 0/2 | Breast cancer  N/A | D  BPs | - | - |
| De Castro et al, 2016^44^ | 2 | 48.5 (46-51) | 0/2 | Osteoporosis | A | Case 1 6y  Case 2 n.s. | Corticosteroids 1  Diabetes 1  Systemic lupus erythematosus 1 |
| Giudice et al, 2020^46^ | 1 | 69 | 1/0 | Osteoporosis | A | 10 y | Hypertension, hypercholesterolemia, former smoker |
| Gönen and Yılmaz Asan, 2017^47^ | 1 | 77 | 1/0 | Prostatic cancer | Z | 2 y | Coronary disease |
| Hao et al, 2022^48^ | 3 | 58.7±4 | 1/2 | Brest cancer 2  Kidney cancer 1 | Z 3 | 25.3±22m  (range 4-48m) | - |
| Law et al, 2021^50^ | 4 | 73±5.2  (68-78) | 0/4 | Breast cancer 1  Osteoporosis 2  Rheumatoid arthritis 1 | Z 1  A 2  I 1 | 2m-15y | - |
| Maluf et al, 2016^51^ | 2 | Case 1, 69  Case 2, 44 | 1/1 | Breast cancer 1  Lung cancer 1 | D 1  B+D 1 | Case 1, 8 m  Case 2, 7 m | - |
| Maluf et al, 2018^52^ | 2 | Case 1, 79  Case 2, 75 | 0/2 | Breast cancer 2 | Z | - | Allergy to penicillin 1  Sjögren’s syndrome 1 |
| Moraes-da-Silva et al, 2023^53^ | 1 | 78 | 0/1 | Osteoporosis | A | 4y | Diabetes, renal dysfunction |
| Pardo-Zamora et al, 2021^54^ | 1 | 72 | 0/1 | Osteoporosis | D LD 1 | D: 2y (4 injections) | - |
| Saad D and Saad P, 2017^55^ | 1 | 64 | 0/1 | Osteoporosis | I+D | I 1y  D 2 y | - |
| Sahin et al, 2019^56^ | 1 | 63 | 0/1 | Osteoporosis | D | 7 y | Hypertension1 |
| Soydan and Uckan, 2014^18^ | 1 | 75 | 1/1 | Multiple myeloma | Z+P | 3 y | Diabetes  Prostate enlargement |
| Tsai et al, 2016^57^ | 1 | 79 | 0/1 | Osteoporosis | Z+A | A 10 y  Z 1 y | - |

Legend

Z: zoledronic acid; P: pamidronic acid; I: ibandronic acid; C: clodronic acid; R: risedronic acid; D: denosumab; S: sunitinib; B: bevacizumab; A: alendronic acid; CR: case reported; n.s.: not specified; BP: bisphosphonate not specified; MBD: metabolic bone disease

Zoledronic acid treatment for cancer patients usually involves administration of 4 mg every 4 weeks

Zoledronic acid treatment for MBD patients usually involves administration of 5 mg once a year

Denosumab treatment for cancer patients usually involves administration of 120 mg every 4 weeks

Denosumab treatment for MBD patients usually involves administration of 60 mg every 6 months

Supplementary Table 6 - L-PRF in the management of MRONJ: specific features and outcomes of case reports

| **Reference** | **Number of lesions** | **Site** | **Staging AAOMS (specified if different)** | **Type of intervention** | **Antibiotic therapy** | **Follow-up** | **Outcome** |
| --- | --- | --- | --- | --- | --- | --- | --- |
| Bouland et al, 2021^41^ | 2 | Maxilla  Mandible | Stage 3  Stage 2 | Surgical treatment + stromal vascular fraction + L-PRF | Amoxicillin 1g 3x/day for 1w | 18m | Complete healing |
| Cortese et al, 2021^42^ | 2 | Mandible | Stage 3 | Surgery + L-PRF | Amox/clav 500mg 3x-7day pre-op and for 4d | 6m | Complete healing |
| De Castro et al, 2016^44^ | 2 | Mandible 2 | Stage 2  1  Stage 3  1 | Surgical debridement + photodynamic therapy (PDT) + L-PRF | Case 1 Amox/clav 1 g + Metronidazole 400 mg x 3/d for 15 d  Case 2 Clindamycin 300 mg x 3/d for 7 d | Case 1 10 m  Case 2 14m | Complete healing |
| Giudice et al, 2020^46^ | 1 | Mandible | Stage 3 | Surgical debridement with piezoelectric device + L-PRF | Amox/clav 1g + Metronidazole 500mg | 5 y | Complete healing |
| Gönen and Yılmaz Asan, 2017^47^ | 1 | Mandible | Stage 3 | Curettage + Sequestrectomy + L-PRF | Amox/clav 1g + metronidazole 500 mg | 18 m | Complete healing |
| Hao et al, 2022^48^ | 3 | Mandible | Stage 3 | Surgery + L-PRF | Amox/clav  Ceftriaxone  Ornidazole | 6m | Complete healing |
| Law et al, 2021^50^ | 4 | Mandible | Stage 2 | Surgical debridement and sequestrectomy + L-PRF | Amox/clav 1g pre-op  Amox/clav 500+125mg for 5-14d | 16.8 m (range 3-36m) | Complete healing 3  No healing 1 |
| Maluf et al, 2016^51^ | 2 | Mandible 2 | Stage 2 | Surgical debridement and sequestrectomy with burs + L-PRF | Penicillin/Clavulanate 875 mg | Case 1 4 m  Case 2 6 m | Partial healing |
| Maluf et al, 2018^52^ | 2 | Mandible 1  Maxilla 1 | Stage 2  Stage 3 | Surgical debridement + L-PRF | Case 1 Amox/clav 1g + metronidazole 250 mg for 1 w  Case 2 Ciprofloxacin 500 mg for 4 w | Case 1 52 m  Case 29 m | Complete healing |
| Moraes-da-Silva et al, 2023^53^ | 3 | Mandible 2  Maxilla 1 | - | Surgical debridement + L-PRF + rhBMP-2 | - | 18m | Complete healing |
| Pardo-Zamora et al, 2021^54^ | 1 | Maxilla | Stage 2 | Surgical debridement and sequestrectomy + L-PRF | Amox/clav 1g 3x/day | 12m | Complete healing |
| Saad D and Saad P, 2017^55^ | 1 | Maxilla | Stage 2 | Surgical debridement and sequestrectomy with piezoelectric device + L-PRF | Amoxicillin/Clavulanic Acid for 7 d | 12 m | Complete healing |
| Sahin et al, 2019^56^ | 1 | Maxilla | - | Surgical debridement and sequestrectomy with piezoelectric device + L-PRF + BFP | Amox/clav 1g + Metronidazole 500mg | 1,3,6,12 m | Complete healing |
| Soydan and Uckan, 2014^18^ | 1 | Maxilla | - | Surgical debridement + L-PRF | Amox/clav 1g + Metronidazole 500mg for 3 w | 6 m | Complete healing |
| Tsai et al, 2016^57^ | 1 | Mandible | Stage 3 | Saucerization and sequestrectomy + L-PRF (GA) | Ciprofloxacin 750 mg/d for 3 m | 10 m | Complete healing |

Legend

Amox/clav: amoxicillin + clavulanate

BFP: Buccal fat pad

GA: under general anesthesia

HBO: hyperbaric oxygen therapy

i.m.: intramuscular administration

i.v.: intravenous administration
